# Thrombotic Sequela of Asymptomatic COVID-19 Infection

**DOI:** 10.7759/cureus.20673

**Published:** 2021-12-25

**Authors:** Zaland A Yousafzai, Wajeeha Qayyum, Qazi Kamran Amin, Ihtesham Shafiq, Nouman Anthony

**Affiliations:** 1 Medicine, Rehman Medical Institute, Peshawar, PAK; 2 General Medicine, Rehman Medical Institute, Peshawar, PAK; 3 Internal Medicine, Rehman Medical Institute, Peshawar, PAK

**Keywords:** ischemic stroke, cerebral infarction, coronavirus, basilar artery, post covid stroke, brain ischemia, sars-cov-2, antinuclear, antibodies, covid-19

## Abstract

Severe coronavirus disease 2019 (COVID-19) is known to be associated with thrombotic events like ischemic stroke. However, in the case of mild or asymptomatic disease, a thrombotic event like ischemic stroke is rare and has never been reported in our country. We present the case of a 28-year-old male patient with no co-morbidities who was diagnosed to have ischemic stroke involving the basilar artery. No risk factors for ischemic stroke could be found except for post-COVID-19 status, evident by the presence of antibodies against severe acute respiratory syndrome coronavirus 2 (SARS-CoV-2).

## Introduction

Coronavirus disease 2019 (COVID-19) is caused by the severe acute respiratory syndrome coronavirus 2 (SARS-CoV-2). It presents with a wide variety of symptoms, the most frequent of which are fever, fatigue, myalgias, dry cough, and in some severe cases, shortness of breath which may progress to respiratory failure and acute respiratory distress syndrome [1]. Thrombotic complications like pulmonary embolism, deep vein thrombosis, ischemic stroke, myocardial infarction, and systemic arterial embolism are well known to be associated with symptomatic and severe disease [2]. Their association with mild or asymptomatic disease has not been mentioned in the literature. As per our knowledge, no cases of ischemic stroke following asymptomatic SARS-CoV-2 infection have been reported from Pakistan. Here we report a case of a basilar artery infarction secondary to asymptomatic COVID-19 in a young male with no co-morbidities.

## Case presentation

 A 28-year-old non-smoker male presented to the emergency department with a sudden loss of consciousness; he had no history of hypertension, diabetes, illicit drug use, recent trauma, fall, fits, or fever. Family history was negative for sudden deaths, early-onset strokes, and cardiovascular disease. On presentation, he was vitally and hemodynamically stable with a blood pressure of 117/73 mmHg, heart rate of 76 bpm, and O_2_ saturation of 98% on room air. On physical examination, his Glasgow Coma Scale (GCS) score was 8/15 (E4 M3 V1). He had decorticate posturing on painful stimuli. Power in all the limbs was hard to assess; deep tendon reflexes were brisk and symmetrical bilaterally, and planters showed extensor response bilaterally. No facial droop or asymmetry was noted, and gag reflex was present. He was completely mute, non-responsive to any command. His eyes were deviated in abducted position. Voluntary movements of eyes were absent in all directions. His pupils were bilaterally dilated and non-reactive to light (≈ 6 mm). Oculocephalic reflex was absent; however, oculovestibular reflex was present.

**Figure 1:**
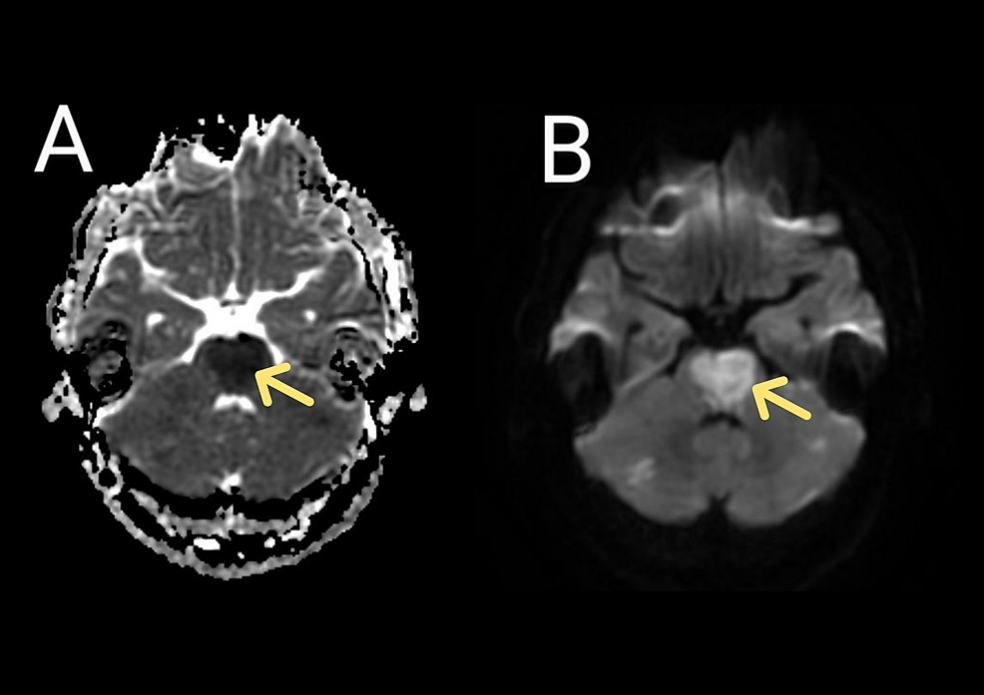
Two scans of magnetic resonance imaging of the brain, yellow arrows point towards the infarct in the brainstem A: Apparent diffusion coefficient (ADC) showing extensive brainstem infarct. B: Diffusion-weighted imaging (DWI) sequence showing brainstem.

**Figure 2:**
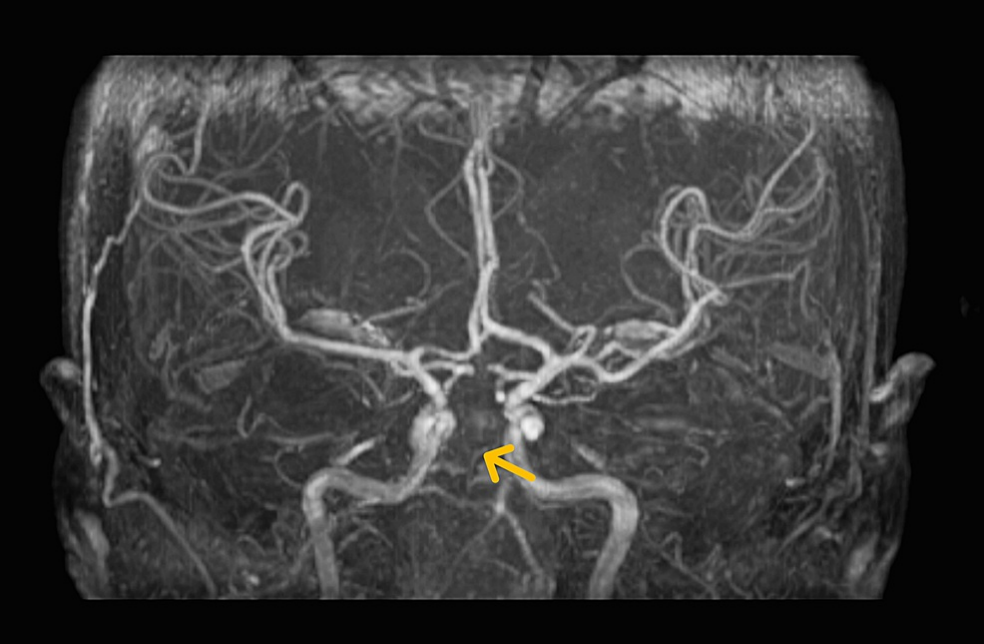
Magnetic resonance angiogram (MRA) of the brain The MRA brain shows the loss of normal flow void in the basilar artery indicated by the yellow arrow.

An MRI of the brain done in the emergency room showed multiple small scattered acute cerebral infarcts affecting the midbrain and pons, bilateral thalami (territory of artery of Percheron), right parietal lobe, central aspect of cerebellar hemisphere along with loss of signal void in the basilar artery (Figure 1). On a magnetic resonance angiogram (MRA) of the brain, loss of normal flow void was noted in the basilar artery (Figure 2). To determine the cause of ischemic stroke in this young male serial investigations were performed, including complete blood counts, C-reactive protein levels, renal and liver function tests, (postprandial) lipid profile, echocardiography, 24-hour Holter monitoring, and carotid doppler (Table 1). Specialized investigations, including a urine toxicology screening, erythrocyte sedimentation rate (ESR), antinuclear antibodies, thyroid function tests, syphilis screening, homocysteine levels, and thrombophilia screening, were done, which turned out to be negative (Table 1). Keeping in view the ongoing COVID-19 pandemic, SARS-CoV-2 antibodies were sent for, which turned out to be high, while the polymerase chain reaction (PCR) test for COVID-19 was negative twice, and no changes were observed on X-ray and CT scan of the chest. The high values of the initial biochemical test were due to dehydration which was corrected with intravenous fluids, and the values were normal on peripheral smear; hence hematologic malignancies and polycythemia were ruled out. A detailed history taken from an immediate relative living with the patient did not reveal any symptoms of the disease in the past, nor had he received any COVID-19 vaccine. After excluding all potential causes for basilar artery thrombosis in this patient, it was attributed to a hypercoagulable state secondary to asymptomatic SARS-COV-2 infection. The patient's condition remained static over the following two weeks, and the patient was shifted to a long-term care facility for future nursing care.

**Table 1:** The patient's laboratory investigation results (with reference values in brackets) Abbreviations: LDL-C: low-density lipoprotein cholesterol, HDL-C: high-density lipoprotein cholesterol, APTT: activated partial thromboplastin time, PT: prothrombin time: INR: international normalized ratio. ESR: erythrocyte sedimentation rate, VDRL: venereal disease research laboratory test.

Investigations	Results
Hemoglobin	17.1 g/dl (12.5–16.5)
Total Leukocyte Count	17.39 x 10^3/uL (4–11)
Platelets	450 x 10^9/L (150–450)
C- reactive Protein	6.45 mg/dl (0.0–0.5)
Total Cholesterol	132 mg/dl (125–200)
LDL-C	86 mg/dl (50–129)
HDL-C	38 mg/dl (40–59)
Triglyceride	158 mg/dl (40–150)
APTT	22.3 sec (Control: 26.0 sec)
PT	10.1 sec (Control: 11.0 sec)
INR	0.92 (0.9–1.3; 0.9–1.3 without anticoagulant, 2.0–3.0 on warfarin therapy)
Anti Nuclear Antibody (ANA)	0.8 (Negative: ≤1.0, Weakly positive: 1.1–2.9, Positive: 3.0–5.9, Strongly positive: ≥6.0 U)
Ionized Calcium	4.73 mg/dl (4.4–5.2)
Magnesium	0.9 mmol/l (0.75–0.95)
Plasma Creatinine	0.8 mg/dl (0.65–1.04)
Plasma Urea	45 mg/dl (10–50)
Sodium	137.4 mmol/l (135–148)
Potassium	3.68 mmol/l (3.6–5.2)
Chloride	101.5 mmol/l (98–108)
Bicarbonate (measured)	24.1 mmol/l (22–28)
Glycated Hemoglobin (HbA1C)	5.3% (<6.5%)
Free Thyroxine (FT4)	14.86 pmol/l (0.7–1.8)
Thyroid Stimulating Hormone (TSH)	1.207 mlU/l (0.46–4.7)
Triiodothyronine (T3)	1.36 nMol/L (1.22–3.07)
​ESR	13 mm/hr (0–15)
Urine toxicology screening	Negative
VDRL (syphilis screening)	Negative
Homocysteine levels	8.6 mcmol/L (<15 micromoles per liter)
Thrombophilia screening: Antithrombin iii, Protein C, Protein S, Factor VIII, Factor V Leiden	Deficiency not detected

## Discussion

Ischemic stroke is a known neurological sequela in patients admitted with COVID-19; a study from China revealed that 5.7% of patients admitted with severe COVID-19 developed cerebrovascular accidents compared with 0.8% of patients with the mild disease [3]. Other studies have also reported ischemic stroke occurring in young individuals with COVID-19, but in both cases, the cerebrovascular accidents were preceded by symptomatic COVID-19 [4,5]. Our patient got a stroke as a sequela of asymptomatic SARS-CoV-2 infection in the past. Comorbidities increase the risk of ischemic stroke after COVID-19 [6]; however, our patient had no comorbidities. A retrospective cohort study from New York reported that COVID-19 patients with high D-dimers had a higher frequency (65.6%) of disabling stroke [7]. Although our patient had a high score on the National Institutes of Health Stroke Scale (NIHSS) on arrival, his D-dimers were normal. A number of mechanisms are proposed for the development of thrombotic events in SARS-CoV-2 infected patients; high D-dimer levels in patients with severe infection can be secondary to exaggerated inflammatory processes termed sepsis-induced coagulopathy (SIC) [8]. The presence of antiphospholipid antibodies has also been described in some cases [9]. Vasculopathy caused by the direct neuroinvasive potential of the virus has also been proposed [6]. In our patient, the mechanism remains unclear. It may be due to damage of vascular endothelium by virus remnants or autoantibodies that led to the thrombus formation leading to stroke.

## Conclusions

It is very unusual to have serious and disabling sequelae of a disease that itself had an asymptomatic course. In our case, treatment options were limited due to the late presentation of the patient with limited facilities. Prophylactic strategy in the setting of asymptomatic infection is also not applicable. These types of case scenarios are such where the physician and patient both are helpless.
